# A Macaque Model of SARS

**DOI:** 10.1371/journal.pmed.0030222

**Published:** 2006-04-18

The 2002 SARS outbreak that started in China spread quickly to Hong Kong, Singapore, Vietnam, and Canada. Although the 774 people it killed was a small number compared with the global death toll from other infectious diseases, the outbreak caused widespread panic because of the lack of global preparedness for what could have become a worldwide epidemic. Since then, surveillance and monitoring systems have been put into place and existing ones strengthened, but since another outbreak is always possible, researchers around the globe are still devoting much time to studying the infection. Analyzing the disease in animals to investigate the pathogenesis of the novel coronavirus that causes SARS (SARS-CoV) is crucial to developing vaccines and treatments to tackle the next epidemic.

In adults, SARS first causes flu-like symptoms, then lower respiratory tract disease, and finally severe respiratory disease. But despite having similar levels of viral replication, children tend to have milder symptoms. They do not get chills or myalgias, nor do they need help breathing, as adults tend to need toward the end of the illness.

Several animals—mice, cats, and ferrets—have been tested to see whether they can support replication of SARS-CoV, and others—civets and wild bats—have been investigated as potential viral reservoirs. In studies on nonhuman primates, the focus has been to document histopathological disease rather than to look for more advanced symptoms such as radiographic evidence of pulmonary disease, as happens in humans.

In a new study, Jason Paragas, James Lawler, and colleagues now describe what happened when they infected eight macaques with the SARS-CoV Urbani strain; four in the nasal cavities and bronchus; two in the nasal cavities and conjunctiva; and two intravenously.

Although all animals had evidence of viral replication and produced neutralizing antibodies, none of the animals developed fever, and only those in the first two groups had mild-to-moderate symptoms (decreased activity and feeding, and slightly labored breathing). By contrast, the animals that had been intravenously infected showed no clinical symptoms. When tested for the presence of the virus, all animals had viral DNA in nasal swabs and urine samples—irrespective of how they had been infected. Paragas and colleagues also took chest radiographs of six of the animals—never before done in any SARS-CoV study on nonhuman primates. Three nonhuman primates showed signs of pneumonia by radiographs.

More interesting findings came from the fact that some animals were infected with wild-type virus, and others with a recombinant infectious clone. All developed similar disease, indicating that it was just the SARS-CoV that was responsible for disease, and that no coinfection was required, as has been suggested by some workers. In addition, six animals that were reinfected with SARS-CoV 13 weeks after the first infection were immune—importantly, two of these had initially had the recombinant virus, which means that the molecular clone could induce protection against the wild-type form.

Paragas and colleagues' work differs from previous studies of SARS-CoV in nonhuman primates. Some researchers found more severe clinical disease; others, no overt disease at all. Tests on African green monkeys showed that one monkey had fever on the third day after infection. These differences could have been because of the strain, the dose, or the route of infection.

Ultimately, disease in nonhuman primates is far milder than that in adult humans. What is interesting is that it is similar to SARS-CoV infection in human children. The researchers suggest that the key to the difference in disease severity could lie in the fact that adult humans with SARS-CoV have far higher levels of inflammatory cytokines than do children, or, as this research suggests, nonhuman primates.

## 

**Figure:**
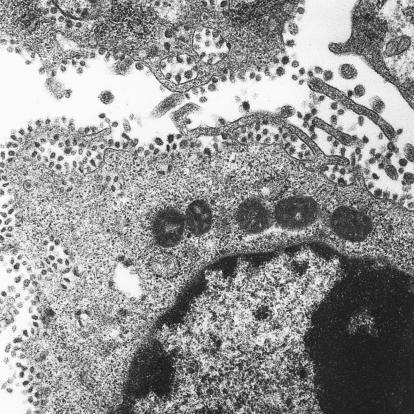
An electron micrograph of SARS-CoV-infected Vero cells

